# Psychological and Behavioral Responses to the COVID-19 Pandemic in Greece

**DOI:** 10.3389/fpsyt.2020.00821

**Published:** 2020-08-19

**Authors:** Eleni Parlapani, Vasiliki Holeva, Panteleimon Voitsidis, Apostolos Blekas, Ioannis Gliatas, Georgia N. Porfyri, Adrianos Golemis, Kalliopi Papadopoulou, Aikaterini Dimitriadou, Aliki F. Chatzigeorgiou, Vasiliki Bairachtari, Sofia Patsiala, Marina Skoupra, Kleoniki Papigkioti, Christina Kafetzopoulou, Ioannis Diakogiannis

**Affiliations:** 1st Department of Psychiatry, School of Medicine, Aristotle University of Thessaloniki, Thessaloniki, Greece

**Keywords:** COVID-19, 2019-nCoV, Fear of COVID-19 Scale, fear, depression, anxiety, social responsibility, compliance

## Abstract

**Objective:**

Fear of COVID-19 was associated with more severe depressive and anxiety symptoms. This study aimed to explore COVID-19-related fear, depressive and anxiety symptoms, social responsibility, and behavioral responses during the COVID-19 pandemic in Greece.

**Method:**

A cross-sectional study was conducted from April 10 to April 13, 2020. Members of the Greek general population completed anonymously an online survey, distributed through the social media. Among the 3,700 adult respondents, 3,029 fulfilled inclusion criteria. The survey included sociodemographic questions, questions exploring potential risk factors for increased fear of COVID-19, questions about the employment of safety and checking behaviors, and questions about compliance with public health guidelines. In addition, four psychometric scales were used, the Fear of COVID-19 Scale (FCV-19S), the Brief Patient Health Questionnaire (PHQ-9) depression scale, the Generalized Anxiety Disorder scale (GAD-7), and Steele’s Social Responsibility Motivation scale. Multivariate General Linear Models (GLM) were used to depict significant differences among dependent variables (FCV-19S, PHQ-9, GAD-7) and independent variables (potential risk factors, safety and checking behaviors, compliance with guidelines). The relationship between the FCV-19S total score and influencing factors was quantified by linear regression analysis.

**Results:**

Several participants reported high levels of COVID-19-related fear (35.7%) and moderate to severe depressive symptoms (22.8%), while a significant proportion reported moderate to severe anxiety symptoms (77.4%). Women scored altogether significantly higher than men. Respondents under the age of 30 reported less fear and depressive symptoms and showed the least social responsibility. Based on GLM, a significant other’s COVID-19 illness, being on psychiatric medication, employment of safety and checking behaviors, and compliance with guidelines were associated with higher COVID-19-related fear. Linear regression analysis revealed that gender, age, depressive, and anxiety symptoms modified levels of COVID-19-related fear.

**Conclusions:**

Greater behavioral responses to the pandemic, that is, excessive employment of safety/checking behaviors and greater compliance with guidelines, were shown to amplify fear, potentially due to increased contamination awareness. In addition, female gender, older age, and more severe anxiety symptoms were related with higher COVID-19-related fear. Describing and weighing carefully the psychosocial and behavioral impact of the pandemic will enable the implementation of both supportive and preventive interventions.

## Introduction

On December 31, 2019, the World Health Organization (WHO) China Country Office received information about the outbreak of a series of pneumonia cases in Wuhan, Hubei, China. The cause was yet unknown, though clinical manifestation resembled pneumonia of a viral origin. A week later, a novel coronavirus, 2019-nCoV, was isolated in China and the information was shared worldwide for the development of diagnostic test kits. WHO announced the emersion of pneumonia in 41 confirmed cases, without specific recommendations for health measures by travelers (1). On January 13, the first COVID-19 case was detected outside China, in Thailand. As a result, WHO recommended that health authorities should raise awareness to limit the spread of respiratory infections through traveling, without suggesting travel/trade restriction measures (2). From there on, the virus started spreading to neighbor Asian countries, to Europe, reaching United States on January 21, when the first confirmed COVID-19 case, a patient who had recently returned from Wuhan, was reported in Washington (3). On January 30, WHO declared COVID-19 a “Public Health Emergency of International Concern” (4). By the end of February, the virus had spread to countries worldwide (5), so that on March 11, WHO declared COVID-19 a pandemic, placing Europe at its center with over 20,000 confirmed cases and as many as 1,000 reported deaths. WHO/Europe advised European countries to prepare for different public health scenarios and encouraged the public to become accustomed, among others, to hand hygiene and social distancing (6).

**Figure 1:**
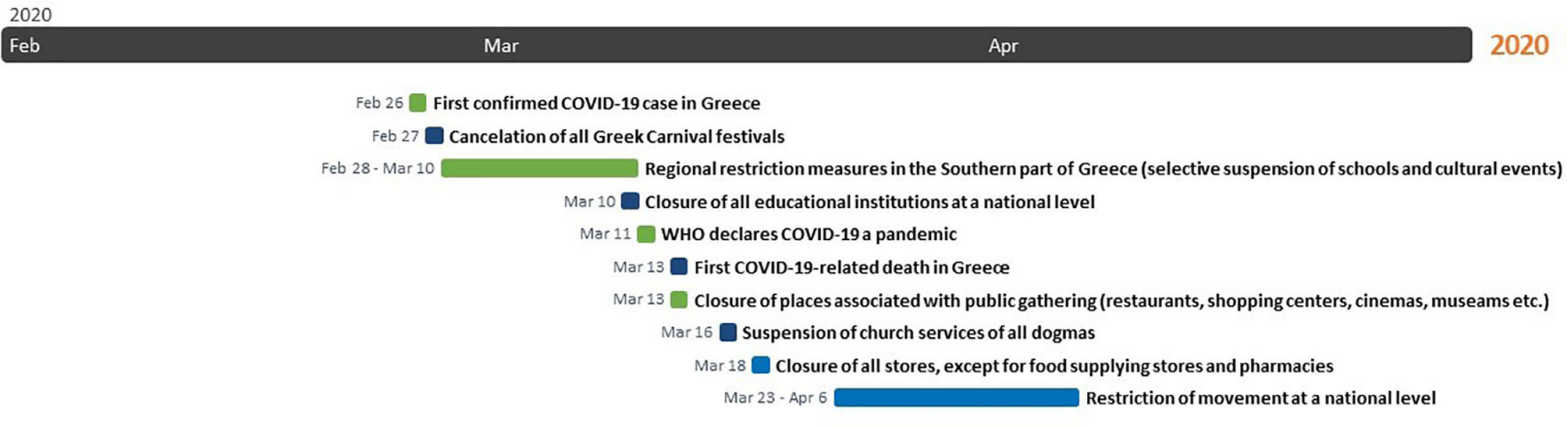
Restriction measures imposed during the COVID-19 pandemic in Greece.

The first confirmed COVID-19 case, a Greek woman who had just returned home from North Italy, was reported on February 26 in Thessaloniki, the second largest city in Greece. As early as next day, the Greek Government started to impose measures against the spread of COVID-19. The Greek Carnival festivals were canceled and restriction measures were taken regionally, mostly in affected areas in the Southern part of Greece, including selective suspension of schools and cultural events. Within a short period and after the COVID-19 cases had risen to 89, the Greek government declared closure of all educational institutions at a national level on March 10. After the first COVID-19-related death had been reported on March 12, the Greek government rapidly escalated restriction measures until March 22, when the Greek Prime Minister announced restriction on movement without cause throughout the country, beginning on March 23 until April 6. The national lockdown was timely imposed, since it was declared as soon as the number of COVID-19 positive cases was 695 and the number of COVID-19-related deaths 17 (**Figure 1**) (7). During the national lockdown, citizens were allowed to leave their house only for specific purposes and after they had filled out a special movement permit handed out by the Greek civil protection or after having texted a designated number, set out for this purpose (8). Moreover, the Hellenic National Public Health Organization (NPHO) released guidelines for proactive controls at incoming and outcoming country sites, that is, airports, harbors, railway stations and road networks (9), as well as for self-quarantine at home for a period of 2 weeks in case of exposure or potential exposure to COVID-19 (10). Two days before the expiring date, restriction measures were extended until April 27, that is, after the Orthodox Easter holiday was over. Consequently, they were further extended until May 4 (11). In summary, in less than a month after the first Greek COVID-19 positive case had been announced, the Greek Government imposed curfew to prevent purposeless movements, restricted traveling and locked city areas and villages down, a novel experience for Greek residents.

Greece has suffered endemics and epidemics before, such as the outbreak of the West Nile virus (2010-2011) and the human immunodeficiency virus (HIV) among injecting drug users (2011). In these cases, although the psychological impact on the general population was not evaluated, it was probably low, since the number of deaths due to the West Nile virus was limited, while the HIV outbreak was confined to a specific subpopulation (12). With regard to the SARS 2003 epidemic, although the Greek hospitals took measures and got prepared to receive cases, the epidemic did not affect Greece (13). With regard to the pandemic influenza A (H1N1), one epidemiologic study provided data related with fatal cases in an effort to enlighten risk factors associated with poor prognosis (14). Within 10 months (May 2009-February 2010), Greece had suffered 140 H1N1-related deaths among 18,075 laboratory-confirmed cases (15). Within 2 months (February 26-April 27), Greece suffered 136 COVID-19-related deaths among 2,534 laboratory confirmed cases (16). Although the psychological impact of H1N1 on the Greek general population was not investigated, a study of healthcare workers revealed that a significant proportion (over half of the participants) were worried about the pandemic, reporting moderately high concern (17). Moreover, the “fear virus” spread by the media was associated with negative attitudes towards H1N1 vaccination, resulting in low compliance rates (18).

Currently, Greece is rather recollecting the experience with the 1918 influenza pandemic or “Spanish flu”, the most severe pandemic crisis in recent history (19), which killed up to one third of the infected population in some areas in Greece (20). During that period, restriction measures had been imposed, including closure of schools, prohibition of pedestrian traffic between 05:00 p.m. and 05.00 a.m. and of any assembly. Violators were arrested immediately (21). A century after the Spanish flu, Greece is reliving strict regulations imposed to restrict the spread of COVID-19. The pandemic disrupted social life, family life, occupational life, education, transportation, traveling, and other aspects of every-day life. It affected Greece during Easter time, the greatest religious holiday of the Orthodox Christian majority of the population, associated with massive celebrations and family reunions throughout the country. Moreover, the future impact on tourism, the spearhead of Greek economy, and finances remain to be seen. According to the World Economic Outlook report released by the International Monetary Fund (IMF), Greece’s unemployment rate is expected to increase by 5 points due to the pandemic, raising from 17.3% to 22.3%. The impact on mental well-being due to the following financial crisis may be prolonged (22).

A pandemic is a public health emergency situation, a life-threatening condition with an impact on community’s normal functioning. Even before a pandemic has reached a country and moreover during the pandemic, a psychological burden is imposed on the general population. Anxiety, fear and uncertainty are common psychological responses to this frightening condition (23). The outbreak of an infectious disease evokes automatically an alert response that may be related with the collective memory of past deadly plagues (24). Fear, embedded in human nature as a result of historical accounts of former frightful epidemics, is amplified by ﬁctional, though vivid dramatizations in the movies (25).

The psychological impact of epidemics on the general population is also associated with the nature of the virus. COVID-19 is an unfamiliar, readily contagious disease associated with mortality. Following the “germ panic” during the 20^th^ century, the COVID-19 pandemic revived the “viral panic” of the 21^st^ century (26). According to a poll of a Canadian (27) and a US population (28), as well as an online survey of a German (29) and a United Kingdom population (30), the COVID-19 pandemic elicited worry. Levels of COVID-19-related fear were positively associated with depressive and anxiety symptoms’ severity (31).

Fear of COVID-19 has different facets. It involves, among others, fear for the body in case of contracting the virus, as well as fear for significant others (32). A cross-sectional study aiming to explore coronavirus-related fear reported that a great proportion of the participants (46.22%) were concerned about the health of friends, grandparents, and loved ones. Moreover, perceived risk of infection and serious illness of loved ones was the strongest predictor of coronavirus-related fear (33). Similarly, a previous study of the psychological impact of H1N1 on Greek healthcare workers reported that most of the participants (60.5%) were worried about their family and friends being infected, as well as about the disease’s dangerousness (54.9%) and consequences on functional ability (43.2%) (17). Lastly, it is expected that people with mental health disorders are likely to be more affected by COVID-19 due to higher vulnerability to stress compared with the general population (23, 34). Taken together, personal experience with COVID-19, COVID-19 illness of significant others, and psychiatric medication intake (indicative of a psychiatric disease’s presence), may be considered as potential risk factors for increased levels of COVID-19-related fear.

From an evolutionary perspective, fear is associated with self-protective responses and therefore risk-avoiding behaviors, promoting self-perseverance (35). In accordance, fear of COVID-19 was related with employment of public health behaviors (30). Compliance with guidelines by health authorities and the government was a remedy for preventing the spread of COVID-19. The last time Greece experienced such a pandemic was a century ago. Since then, strict restriction measures to avoid spread of a virus had never been necessary.

Up to date, the impact of infectious disease outbreaks on mental health has not been investigated in the Greek general population. Based on experience from the 2002–2003 SARS epidemic, socio-cultural factors influence the psychological impact of a pandemic (36, 37). Moreover, the members of the general population may demonstrate different responses during a crisis based on age, gender, educational status and other factors. Therefore, the main aim of the present study was to explore the psychological responses, that is, fear of COVID-19, depressive and anxiety symptoms during the COVID-19 pandemic in Greece, as well as to highlight factors that may modulate severity of the COVID-19-related psychological impact. Secondary aims were to explore three potential risk factors for increased levels of COVID-19-related fear (personal experience with COVID-19; COVID-19 illness of significant others; psychiatric medication intake, indicative of a psychiatric disease’s presence), to investigate behavioral responses (safety behaviors; checking behaviors), as well as to assess compliance with public health guidelines and social responsibility motivation. Lastly, the association between psychological and behavior responses was examined.

## Methods

### Participants

A cross-sectional study was conducted from April 10 until April 13, 2020, that is, 3 weeks after the national lockdown measures had been imposed, *via* an online survey. The survey was distributed through the social media (e.g. Facebook, LinkedIn). Members of the Greek general population were invited to join the study voluntarily. Potential participants accessing the survey platform were informed about the nature of the research and data usage. Before entering the survey, respondents were requested to indicate their consent by ticking the consent checkbox, a required field. The questionnaires were filled out anonymously to protect participants’ personal data. In order to secure anonymity, the setting “anonymize responses” was enabled while creating the survey on the online platform; enabling this setting prevented recording of any personal information and permanently removed contact association from the results, before saving in the data (38). Inclusion criteria were: i) acceptance to participate; ii) being adult; iii) completion of over 96% of survey questions, a setting enabled while creating the online survey to obtain adequately filled out surveys (38).

Out of the 3,700 responded surveys obtained within the 3-day study period, 671 were excluded because they did not fulfill the criterion of 96% completion. Therefore, 3,029 participants entered the study (81.86% completion rate).

Ethical approval was received from the Scientific Committee of the General Hospital “Papageorgiou” Review Board prior to data collection.

### Survey Design

At first, the survey questions and scales were selected based on author’s experience and available literature on pandemics. After selection and synthesis of the survey’s material, it was significant to investigate whether participants could perceive the survey’s scope, as well as to detect potential difficulties encountered during questions’ completion. Consequently, a survey sample was distributed to six mental health professionals, three psychiatrists and three psychologists during the pre-survey phase. Upon completion of the material, professional’s feedback contributed to appropriate modifications prior to survey’s web publication. Conciseness was achieved through removal of redundant and irrelevant queries that could bring confusion. Questions were placed in a logical sequence to provide a better mental roadmap while filling out the survey (39).

The final survey format, established after all necessary changes had been applied, included a total of 113 queries in Greek. The time required for survey completion was estimated at around 15 min. The online survey platform was created using the Qualtrics platform (40).

### Measures

The survey included a) basic sociodemographic questions, including age, gender, place of residence (urban, small city, rural), living status (alone, with family, with significant others), educational level (elementary, high school 3 or 6 years, Master degree, PhD) and employment status (employed versus unemployed); b) questions exploring three potential risk factors (PRF) for increased fear of COVID-19, three types of safety behaviors (SB), three types of checking behaviors (ChB), as well as compliance (Comp) with the WHO and the Greek government’s guidelines (**Table 1**); c) psychometric scales assessing fear of COVID-19, depression, anxiety and social responsibility, namely:

1. The Fear of COVID-19 Scale (FCV-19S), a reliable and valid unidimensional scale for assessing COVID-19-related fear (31) that was recently developed to support management of the COVID-19 pandemic. This is a 7-item self-report tool, measuring fear of COVID-19 (e.g. item 1, “I am most afraid of coronavirus-19”; item 4, “I am afraid of losing my life because of coronavirus-19”; item 7, “My heart races or palpitates when I think about getting coronavirus-19”) based on a 5-point scale (1 = strongly disagree, 2 = disagree, 3 = neither agree nor disagree, 4 = agree, 5 = strongly agree). Total scores range between 7 and 35. Higher scores reflect greater fear of COVID-19. The current study applied the Greek version of FCV-19S, which demonstrated a very good reliability (Cronbach’s alpha coefficient based on standardized items was 0.87), good concurrent validity [high statistical significant correlation between FCV-19S and GAD-7 (r = 0.71, p <.001); moderate correlation between FCV-19S and PHQ-9 (r= 0.47, p <.001)], as well as good fit indices (RMSE = 0.11; 90% CI = [0.10, 0.11]; CFI = 0.89; TLI = 0.83; and SRMR = 0.06). The cutoff score equal or above 19 indicated high levels of COVID-19-related fear (41, 42).
2. The Brief Patient Health Questionnaire (PHQ-9) depression scale, the 9-item depression module from the complete Patient Health Questionnaire (PHQ) (43), is a self-administered tool for the diagnosis of both major depression and subthreshold depression in the general population (44). The scale assesses symptoms’ severity over the past 2 weeks. Items (e.g. item 1, “Little interest or pleasure in doing things”) are scored based on a 4-point severity scale (0 = not at all, 1 = several days, 2 = more than half the days, 3 = nearly every day). Total scores range between 0 and 27, with higher scores indicating more severe depressive symptoms. The cutoff point of 10 or greater corresponds to moderate to severe depressive symptoms, potentially indicating a clinically significant condition (cutoff scores: 0–4 = minimal or none; 5–9 = mild; 10–14 = moderate; 15–19 = moderately severe; 20–27 = severe). The current study applied the Greek version of PHQ-9 (45, 46).
3. The Generalized Anxiety Disorder scale (GAD-7), initially developed for the assessment of generalized anxiety disorder, is a useful self-report anxiety scale, assessing symptoms’ severity over the past 2 weeks (47). Each of the seven items (e.g. item 1, “Feeling nervous, anxious or on edge”) is scored based on a 4-point severity scale (0 = not at all, 1 = several days, 2 = more than half the days, 3 = nearly every day). Total scores range between 0 and 21. Higher scores indicate more severe anxiety symptoms. The cutoff point of 10 or greater corresponds to moderate to severe anxiety symptoms, potentially indicating a clinically significant condition (cutoff scores: 0–5 = mild; 6–10 = moderate; 11–15 = moderately severe; 15–21 = severe). The current study applied the Greek version of GAD-7 (45, 48).
4. The Social Responsibility Motivation Scale is a four-item tool applied as a proxy measure for motivation to undertake social responsibility (49). Items (e.g. item 2, “I believe that I have a responsibility to help others”) are scored based on a 5-point scale (1 = not at all important; 5 = very important). Total scores range between 4 and 20. Higher scores indicate increased social responsibility motivation. The psychometric properties of the scale’s Greek version indicated adequate reliability (Cronbach’s alpha = 0.70).

**Table 1:** Survey questions exploring potential risk factors for increased fear of COVID-19, safety behaviors, checking behaviors, and compliance with guidelines.

Questions	Possible answers
**Potential Risk Factors (PRF):**	
Have you contracted the virus? (PRF1)	Yes/No/I don’t know
Has someone close to you contracted the virus? (PRF2)	Yes/No/I don’t know
Have you been on psychiatric medication during the past 6 months? (PRF3)	Yes/No
**Safety Behaviors (SB):**	
I clean/disinfect the objects that I use (SB1)	Never/Rarely/Often/Always
I take care of my personal hygiene (e.g. washing my hands) (SB2)	Same as before/According to NPHO/Excessively
I use personal protective equipment (e.g. face masks, disposable gloves) (SB3)	Never/Rarely/Often/Always
**Checking Behaviors (ChB):**	
I check myself for COVID-19 symptoms (e.g. using thermometer) (ChB1)	Never/Rarely/Some days/Daily
I have restricted physical contact with other people (kisses, hugs, sex, and handshakes) (ChB2)	Yes/No
I communicate with my family doctor because I think I have COVID-19 (ChB3)	Never/Rarely/Often
**Compliance with guidelines (Comp):**	
I follow the instructions of the World Health Organization (Comp1)	Never/Rarely/Often/Always
I abide by the measures that the government has enacted to avoid spread of COVID-19 (Comp2)	Never/Rarely/Often/Always

NPHO, National Public Health Organization.

### Procedures

All participants answered questions and completed scales in the following order: sociodemographic data, potential risk factors for increased fear of COVID-19, safety behaviors, checking behaviors, social responsibility motivation scale, compliance with WHO and government’ guidelines, FCV-19S, PHQ-9 and GAD-7. A short introduction was provided before every blog of questions, informing the participants about the questionnaire’s scope. Respondents were asked to click on the circle corresponding to their chosen answer. All questions were single answer questions. Smart branching was also used.

### Statistical Analysis

Data and parameter estimates were presented as mean values, standard deviations (SD), or numbers and percentages. The relationship between categorical variables was assessed by contingency tables and chi-square statistics. Assumptions of homoscedasticity and multicollinearity were checked before further analysis, and when significant interactions appeared between the variables, the file was split and only the significant findings were reported. Whenever Levene’s test for homogeneity of variance was significant at the p level non parametric tests were used to confirm the results (parametric tests reported). Multivariate General Linear Models (GLM) were used to depict significant differences among dependent variables (FCV-19S, PHQ-9, GAD-7) and independent variables (potential risk factors, safety and checking behaviors, compliance with guidelines). The relationship between the FCV-19S total score and influencing factors (gender, age, PHQ-9, GAD-7) was quantified by linear regression analysis. All analyses were performed by the Statistical Package for Social Sciences (SPSS) version 26 (50).

## Results

### Sociodemographic Characteristics

Among a total of 3,029 study participants, 2,177 were female (71.9%) and 737 were male (24.3%). The remaining 115 (3.8%) did not specify gender.

Although women’s age (M_age_ = 34.99, SD = 12.50) was slightly lower than men’s (M_age_ = 35.14, SD = 12.87), the difference was not statistically significant (p >.001).

The majority of the participants had a University Degree [N = 1,360 (68.3%)], and lived in an urban area [N = 2,298 (76.3%)] with their family or significant other [N = 2,538 (83.8%)].

Lastly, the unemployment rate was 14.2% (426 subjects).

### Potential Risk Factors for Increased Fear of COVID-19

Among participants, 0.4% reported that they had contracted the virus, whereas 22.8% reported that they weren’t sure. Another 2.3% of the sample had someone close to them that was infected by COVID-19, whereas 15.8% were uncertain. Lastly, only 8.3% of the respondents were on psychiatric medication during the past 6 months. Frequencies of the potential risk factors for increased fear of COVID-19 and their association with age are presented in **Table 2**.

**Table 2:** Potential risk factors for increased fear of COVID-19 (PRF) and age.

		PRF1: Have you contracted the virus?			Total n (%)	Chi square tests of independence
		Yes n (%)	No n (%)	I don’t know n (%)		
Age	18–30	4 (0.3%)	1160 (75.4%)	374 (24.3%)	1538 (100%)	x^2^(8) = 8.40 p = .395 V_cramer_ = .38 (ns)
	31–45	3 (0.4%)	586(78.3%)	159 (21.3%)	748 (100%)	
	46–60	2 (0.4%)	421 (77.4%)	121 (22.2%)	544 (100%)	
	61–75	0 (0%)	82 (82%)	18 (18%)	100 (100%)	
	> 75	0 (0%)	11 (100%)	0 (100%)	11 (100%)	
Total (PRF1)		9 (0.4%)	2260 (76.8%)	672 (22.8%)	2941 (100%)	
		**PRF2: Has someone close to you contracted the virus?**			**Total** **n (%)**	**Chi square tests of independence**
		**Yes** **n (%)**	**No** **n (%)**	**I don’t know** **n (%)**		
Age	18–30	41 (2.6%)	1273 (82.0%)	238 (15.3%)	1552 (100%)	x^2^(8) = 10.24 p = .248 V_cramer_ = .04 (ns)
	31–45	14 (1.8%)	616 (80.8%)	132 (17.3%)	762 (100%)	
	46–60	9 (1.6%)	455 (82.1%)	90 (16.2%)	554 (100%)	
	61–75	3 (2.9%)	88 (86.3%)	11 (10.8%)	102 (100%)	
	> 75	1 (9.1%)	10 (9.1%)	0 (0%)	11 (100%)	
Total (PRF2)		68 (2.3%)	2442 (81.9%)	471 (15.8%)	2981 (100%)	
		**PRF3: Have you been on psychiatric medication** **during the past 6 months?**			**Total** **n (%)**	**Chi square tests of independence**
		**Yes** **n (%)**	**No** **n (%)**			
Age	18–30	74 (4.7%)	1485 (95.3%)		1559 (100%)	x^2^(4) = 8.40 p <.001 V_cramer_ = .15
	31–45	73 (9.5%)	694 (90.5%)		767 (100%)	
	46–60	81 (14.6%)	473 (85.4%)		554 (100%)	
	61–75	19 (18.6%)	83 (81.4%)		102 (100%)	
	> 75	0 (0%)	11 (100%)		11 (100%)	
Total (PRF3)		247 (8.3%)	2746 (91.7%)		2993 (100%)	

### Safety Behaviors

The majority of the sample (49.8%) reported that they often clean or disinfect the objects they are using, while some reported that they do it always (29.8%). The majority of the respondents (62.4%) followed the NPHO guidelines when it comes to taking care of personal hygiene, but some (17.3%) acknowledged that they may overdo it. Lastly, 37.4% of the respondents stated that they often use protective means, whereas 33.2% reported that they rarely use it. Safety behaviors and their association with age are presented in **Table 3**.

**Table 3:** Safety behaviors (SB) and age.

		SB1: I clean/disinfect the objects that I use				Total n (%)	Chi square tests of independence
		Never n (%)	Rarely n (%)	Often n (%)	Always n (%)		
Age	18–30	54 (3.5%)	272 (17.5%)	787 (50.7%)	440 (28.3%)	1553 (100%)	x^2^(12) = 36.27 p <.001 V_cramer_ = .06
	31–45	29 (3.8%)	135 (17.8%)	386 (59.9%)	209 (27.5%)	759 (100%)	
	46–60	18 (3.3%)	81 (14.6%)	254 (45.9%)	200 (36.2%)	553 (100%)	
	61–75	2 (2%)	12 (11.9%)	51 (50.5%)	36 (35.6%)	101 (100%)	
	> 75	3 (27.3%)	1 (9.1%)	4 (36.4%)	3 (27.3%)	11 (100%)	
Total (SB1)		106 (3.6%)	501 (16.8%)	1482 (49.8%)	888 (29.8%)	2977 (100%)	
		**SB2: I take care of my personal hygiene**				**Total** **n (%)**	**Chi square tests of independence**
		**Same as before** **n (%**)	**According to NPHO** **n (%)**	**Excessively** **n (%)**			
Age	18–30	385 (25,9%)	883 (57.4%)	270 (17.6%)		1538 (100%)	x^2^(8) = 70.62 p <.001 V_cramer_ = .11
	31–45	145 (19.4%)	491 (65.6%)	112 (15%)		748 (100%)	
	46–60	55 (10.2%)	379 (70.4%)	104 (19.3%)		538 (100%)	
	61–75	8 (8.7%)	68 (73.9%)	16 (17.4%)		92 (100%)	
	> 75	2 (22.2%)	4 (44%)	3 (33.3%)		9 (100%)	
Total (SB2)		595 (20.3%)	1825 (62.4%)	505 (17.3%)		2925 (100%)	
		**SB3: I use personal protective equipment**				**Total** **n (%)**	**Chi square tests of independence**
		**Never** **n (%)**	**Rarely** **n (%)**	**Often** **n (%)**	**Always** **n (%)**		
Age	18–30	290 (18.7%)	535 (34%)	558 (35.9%)	171 (11.0%)	1554 (100%)	x^2^(12) = 57.96 p <.001 V_cramer_ = .81
	31–45	98 (12.9%)	240 (31.6%)	303 (39.9%)	119 (15.7%)	760 (100%)	
	46–60	68 (12.3%)	176 (31.9%)	209 (37.9%)	99 (17.9%)	552 (100%)	
	61–75	8 (8.0%)	36 (36.0%)	43 (43.0%)	13 (13%)	100 (100%)	
	> 75	5 (45.5%)	2 (18.2%)	0 (0%)	4 (36.4%)	11 (100%)	
Total (SB3)		469 (15.8)	989 (33.2%)	1113 (37.4%)	406 (13.6%)	2977 (100%)	

### Checking Behaviors

With regard to checking behaviors, 44.9% of the respondents did not check themselves for COVID-19 symptoms, whereas 4.5% checked themselves daily. The vast majority (88.4%) restricted physical contact with other people, whereas 2.6% communicated often with their doctor because they were afraid of being sick with COVID-19. Checking behaviors and their association with age are presented in **Table 4**.

**Table 4:** Checking behaviors (ChB) and age.

		ChB1: I check myself for COVID-19 symptoms				Total n (%)	Chi square tests of independence
		Never n (%)	Rarely n (%)	Some days n (%)	Daily n (%)		
Age	18–30	655 (42.1%)	505 (32.4%)	340 (21.8%)	57 (3.7%)	1557 (100%)	x^2^(12) = 26.99 p = .009 V_cramer_ = .55
	31–45	351 (46.1%)	232 (30.5%)	142 (18.7%)	36 (4.7%)	761 (100%)	
	46–60	275 (49.7%)	144 (26.0%)	102 (18.4%)	32 (5.8%)	553 (100%)	
	61–75	50 (50%)	24 (24.0%)	18 (18%)	8 (8.0%)	100 (100%)	
	> 75	7 (63.6%)	2 (18.2%)	2 (18.2%)	0 (0%)	11 (100%)	
Total (ChB1)		1338 (44.9%)	907 (30.4%)	604 (20.3%)	133 (4.5%)	2982 (100%)	
		**ChB2: I have restricted physical contact with other people**				**Total** **n (%)**	**Chi square tests of independence**
		**Yes** **n (%)**	**No** **n (%)**				
Age	18–30	1333 (85.9%)	218 (14.1%)			1551 (100%)	x^2^(12) = 33.90 p <.001 V_cramer_ =.10
	31–45	668 (88.5%)	87 (11.5%)			755 (100%)	
	46–60	508 (92.9%)	39 (7.1%)			547 (100%)	
	61–75	97 (97%)	0 (0%)			97 (100%)	
	> 75	11 (100%)	0 (0%)			11 (100%)	
Total (ChB2)		2617 (88.4%)	344 (11.6%)			2961 (100%)	
		**ChB3: I communicate with my family doctor** **because I think I have COVID-19**				**Total** **n (%)**	**Chi square tests of independence**
		**Never** **n (%)**	**Rarely** **n (%)**	**Often** **n (%)**			
Age	18–30	1387 (89.1%)	124 (8.0%)	46 (3.0%)		1557 (100%)	x^2^(12) = 14.26 p >.075 V_cramer_ = .04
	31–45	695 (90.6%)	48 (6.3%)	24 (3.1%)		767 (100%)	
	46–60	517 (93%)	32 (5.8%)	7 (1.3%)		556 (100%)	
	61–75	96 (94.1%)	6 (5.9%)	0 (0%)		102 (100%)	
	> 75	11 (100%)	0 (0%)	0 (0%)		11 (100%)	
Total (ChB3)		2706 (90.4%)	210 (7.0%)	77 (2.6%)		2993 (100%)	

### Compliance With Guidelines and Social Responsibility

Half of the participants stated that they always follow both WHO instructions (50.6%) and the Greek government’s enacted measures (50.6%). Compliance and its association with age is presented in **Table 5**.

**Table 5:** Compliance with guidelines (Comp) and age.

		Comp1: I follow the instructions of the World Health Organization				Total n (%)	Chi square tests of independence
		Never n (%)	Rarely n (%)	Often n (%)	Always n (%)		
Age	18–30	22 (1.4%)	108 (7.0%)	724 (46.6%)	698(45.0%)	1552 (100%)	x^2^(12) = 75.13 p <.001 V_cramer_ = .09
	31–45	2 (0.3%)	35 (4.6%)	334 (43.8%)	392 (51.4%)	763 (100%)	
	46–60	3 (0.5%)	20 (3.6%)	189 (34.2%)	341 (61.71%)	553 (100%)	
	61–75	0 (0%)	0 (0%)	32 (31.4%)	70 (68.6%)	102 (100%)	
	> 75	0 (0%)	0 (0%)	4 (36.4%)	7 (63.6%)	11 (100%)	
Total (Comp1)		27 (0.9%)	163 (5.5%)	1283(43.0%)	1508 (50.6%)	2981 (100%)	
		**Comp2: I abide by the measures that the government has enacted to avoid spread of COVID-19**				**Total** **n (%)**	**Chi square tests of independence**
		**Never** **n (%)**	**Rarely** **n (%)**	**Often** **n (%)**	**Always** **n (%)**		
Age	18–30	8 (0.5%)	38 (2.4%)	533 (34.2%)	980(50.0%)	1559 (100%)	x^2^(12) = 95.68 p <.001 V_cramer_ = .10
	31–45	3 (0.4%)	6 (0.8%)	211 (27.5%)	547 (51.4%)	767 (100%)	
	46–60	0 (0%)	3 (0.5%)	99 (17.8%)	454 (61.7%)	556 (100%)	
	61–75	3 (2.9%)	1 (1.0%)	20 (19.6%)	78 (76.5%)	102 (100%)	
	> 75	0 (0%)	0 (0%)	3 (27.3%)	8 (72.7%)	11 (100%)	
Total (Comp1)		14 (0.9%)	48 (5.5%)	866(43.0%)	2067 (50.6%)	2995 (100%)	

Subjects aged 18–30 showed less social responsibility. Specifically, *post hoc* analyses using the Bonferroni criterion indicated that social responsibility in this age category (Msrms = 16.09, SD = 2.12) was significantly lower [F(4,2558) = 5866, p <.001] than the age categories 31-45 (M = 16.30, SD = 2.07), 46–60 (M = 16.55, SD = 2.06), 46–60 (M = 16.55, SD = 2.06), 61–75 (M = 16.61, SD = 1.79), and over 75 (M = 16.55, SD = 2.10).

### Psychometric Scales

Severity of fear of COVID-19, depressive and anxiety symptoms was categorized based on the proposed cutoff scores of the continuous scales FCV-19S, PHQ-9, and GAD-7. A significant proportion of the participants (35.7%) reported high levels of COVID-19 fear and severe anxiety symptoms (36.7%). Only a few (0.4%) reported severe depressive symptoms. Female participants had the highest representation in the more severe categories with a statically significant difference (**Table 6**).

**Table 6:** Participants’ categorization based on FCV-19S, PHQ-9, and GAD-7 cutoff scores.

		Total		Male		Female		p
		n	%	n	%	n	%	
FCV-19S	Normal fear	1936	64.3	585	79.4	1302	59.8	x^2^ = 92.38, df = 1, p = .001
	High fear	1074	35.7	152	20.6	875	40.2	
PHQ-9	Minimal-none	1079	35.9	384	52.1	660	30.3	x^2^ = 92.38, df = 1, p = .001
	Mild	1241	41.3	264	35.8	942	43.3	
	Moderate	538	17.9	74	10.0	444	20.4	
	Moderately severe	136	4.5	13	1.8	118	5.4	
	Severe	13	0.4	2	0.3	11	0.5	
GAD-7	Mild	662	22.6	262	36.2	381	17.9	x^2^ = 92.38, df = 1, p = .001
	Moderate	1195	40.7	313	43.3	848	39.9	
	Moderately severe	0	0	0	0	0	0	
	Severe	1077	36.7	148	20.5	894	42.1	

FCV-19S, Fear of COVID-19 Scale (cutoff scores: normal fear < 19; high fear ≥ 19); PHQ-9, Brief Patient Health Questionnaire Depression Scale (cutoff scores: minimal-none = 0–4; mild = 5–9; moderate = 10–14; moderately severe = 15–19; severe = 20–27); GAD-7, Generalized Anxiety Disorder scale (cutoff scores: mild = 0–5; moderate = 6–10; moderately severe = 11–15; severe = 15–21).

As expected, FCV-19S, PHQ-9, and GAD-7 demonstrated significant concurrent correlations. Females reported higher levels of COVID-19-related fear and reported more severe depressive and anxiety symptoms compared with men. Younger respondents (aged 18–30) reported less fear and depressive symptomatology than the other age categories, but they did not present any differences with regard to anxiety symptoms. There were no significant correlations between other sociodemographic characteristics and the psychometric scales (**Table 7**).

**Table 7:** Descriptive statistics and correlations of key variables.

Variables	*1*	*2*	*3*	*Age*	*Gender*	
FCV-19S	–			F(4,2952) = 21.96, p <.001, η^2^ = 0.05	Male: (14.69 ± 4.98) Female: (17.43 ± 5.09)	t_(2878)_ = -12.63 p <.001 η^2^ = 0.05
PHQ-9	.47**	–		F(4,2988) = 13.02, p <.001, η^2^ = 0.05	Male: (12.99 ± 3.90) Female: (15.26 ± 4.57)	t_(1471)_ = -13.07 p <.001 η^2^ = 0.02
GAD-7	.71**	.76**	–	F(4,3286) = 1.51, p = .185, η^2^ = 0.06	Male: (11.25 ± 4.12) Female: (13.89 ± 4.62)	t_(1386)_ = -14.3 p <.001 η^2^ = 0.05
Mean	16.77	14.70	13.23			
SD	5.23	4.51	4.66			

FCV-19S, Fear of COVID-19 Scale; PHQ-9, Brief Patient Health Questionnaire Depression scale; GAD-7, Generalized Anxiety Disorder scale.

The social responsibility scale presented neither a significant correlation with the other psychometric scales (p >.001) nor significant differences between males and females.

### Linear Associations

A multivariate linear model was conducted to explore the relations between potential risk factors, safety behaviors, checking behaviors, compliance with guidelines and the psychometric scales. In testing our hypotheses through GLM modeling, fear of COVID-19 (assessed by FCV-19S), depressive symptoms (assessed by PHQ-9) and anxiety symptoms (assessed by GAD-7) were found to be significantly associated with every dependent variable tested (p <.001) in all but one case. The main effect for ChB2 was not significant [F(3, 2012) = .52, p = .672, η^2^ = .00], suggesting that the linear combination of FCV-19S, PHQ-9, and GAD-7 was similar for each level of ChB2 (**Tables 8** and **9**).

**Table 8:** Multivariate General Linear Model.

Variable	Pillai’s Trace	F	df	SE	p	η^2^
Intercept	.16	171.43^b^	3	2640	<.001	.16
Gender	.05	49.89^b^	3	2640	<.001	.05
Age	.06	60.16^b^	3	2640	<.001	.06
PRF1	.00	2.14	6	5282	.045	.00
PRF2	.00	2.30	6	5282	.032	.00
PRF3	.02	19.04^b^	3	2640	<.001	.00
SB1	.01	4.93	9	7926	<.001	.02
SB2	.03	14.95	6	5282	<.001	.00
SB3	.01	4.34	9	7926	<.001	.01
ChB1	.03	9.59	9	7926	<.001	.00
ChB2	.00	1.16^b^	3	2640	.323	.01
ChB3	.01	6.01	6	5282	<.001	.00
Comp1	.00	2.56	9	7926	.006	.00
Comp2	.01	3.43	9	7926	<.001	.00

^a^Design: Intercept + Gender + Age + PRF1 + PRF2 + PRF3 + SB1 + SB2 + SB3 + ChB1 + ChB2 + ChB3 + Comp1 + Comp2; ^b^Exact statistic; PRF1: Have you contracted the virus; PRF2: Has someone close to you contracted the virus; PRF3: Have you been on psychiatric medication during the past 6 months; SB1: I clean/disinfect the objects that I use; SB2: I take care of my personal hygiene; SB3: I use personal protective equipment; ChB1: I check myself for COVID-19 symptoms; ChB2: I have restricted physical contact with other people; ChB3: I communicate with my family doctor because I think I have COVID-19; Comp1: I follow the instructions of the World Health Organization; Comp2: I abide by the measures that the government has enacted to avoid spread of COVID-19.

**Table 9:** Between subjects effects.

Source	Dependent Variable	SS	df	Mean Square	F	p	η^2^
Corrected Model	FCV-19S	18459.76^a^	27	683.69	33.94	<.001	.258
	PHQ-9	6968.83^b^	27	258.10	14.30	<.001	.128
	GAD-7	10784.32^c^	27	399.42	22.56	<.001	.187
Intercept	FCV-19S	5708.78	1	5708.78	283.39	<.001	.097
	PHQ-9	6952.90	1	6952.90	385.44	<.001	.127
	GAD-7	3385.56	1	3385.56	191.22	<.001	.067
Gender	FCV-19S	1916.79	1	1916.79	95.153	<.001	.035
	PHQ-9	2122.59	1	2122.59	117.67	<.001	.043
	GAD-7	2128.42	1	2128.42	120.21	<.001	.044
Age	FCV-19S	985.09	1	985.09	48.90	<.001	.018
	PHQ-9	864.28	1	864.28	47.91	<.001	.018
	GAD-7	78.81	1	78.81	4.45	.035	.002
PRF1	FCV-19S	60.46	2	30.23	1.50	.223	.001
	PHQ-9	64.47	2	32.23	1.78	.168	.001
	GAD-7	15.56	2	7.78	.44	.644	.000
PRF2	FCV-19S	236.10	2	118.05	5.86	.003	.004
	PHQ-9	40.25	2	20.12	1.11	.328	.001
	GAD-7	128.16	2	64.08	3.61	.027	.003
PRF3	FCV-19S	84.58	1	84.58	4.19	.041	.002
	PHQ-9	970.77	1	970.77	53.81	<.001	.020
	GAD-7	554.34	1	554.34	31.31	<.001	.012
SB1	FCV-19S	563.82	3	187.94	9.33	<.001	.010
	PHQ-9	66.86	3	22.28	1.23	.295	.001
	GAD-7	170.49	3	56.83	3.21	.022	.004
SB2	FCV-19S	1603.20	2	801.60	39.79	<.001	.029
	PHQ-9	598.49	2	299.24	16.58	<.001	.012
	GAD-7	1221.37	2	610.68	34.49	<.001	.025
SB3	FCV-19S	440.68	3	146.89	7.29	<.001	.008
	PHQ-9	183.05	3	61.01	3.38	.017	.004
	GAD-7	136.18	3	45.39	2.56	.053	.003
ChB1	FCV-19S	1502.30	3	500.76	24.85	<.001	.027
	PHQ-9	399.32	3	133.10	7.37	<.001	.008
	GAD-7	1141.84	3	380.61	21.49	<.001	.024
ChB2	FCV-19S	41.81	1	41.81	2.07	.150	.001
	PHQ-9	52.94	1	52.94	2.93	.087	.001
	GAD-7	43.02	1	43.02	2.43	.119	.001
ChB3	FCV-19S	583.07	2	291.53	14.47	<.001	.011
	PHQ-9	190.58	2	95.29	5.28	.005	.004
	GAD-7	123.90	2	61.95	3.49	.030	.003
Comp1	FCV-19S	163.55	3	54.51	2.70	.044	.003
	PHQ-9	35.29	3	11.76	.65	.581	.001
	GAD-7	56.40	3	18.80	1.06	.364	.001
Comp2	FCV-19S	284.62	3	94.87	4.71	.003	.005
	PHQ-9	8.33	3	2.77	.15	.927	.000
	GAD-7	164.42	3	54.80	3.09	.026	.004

^a^R^2^ = .258 (Adjusted R^2^ = .250); ^b^R^2^ = .128 (Adjusted R^2^ = .119); ^c^R^2^ = .187 (Adjusted R^2^ = .179); PRF1: Have you contracted the virus; PRF2: Has someone close to you contracted the virus; PRF3: Have you been on psychiatric medication during the past 6 months; SB1: I clean/disinfect the objects that I use; SB2: I take care of my personal hygiene; SB3: I use personal protective equipment; ChB1: I check myself for COVID-19 symptoms; ChB2: I have restricted physical contact with other people; ChB3: I communicate with my family doctor because I think I have COVID-19; Comp1: I follow the instructions of the World Health Organization; Comp2: I abide by the measures that the government has enacted to avoid spread of COVID-19.

A linear regression analysis was conducted to assess whether gender, age, PHQ-9, and GAD-7 significantly predicted FCV-19S. The “Enter” variable selection method was chosen for the linear regression model, which included the selected predictors. Assumptions of homoscedasticity and multicollinearity were met and all predictors in the regression model presented VIFs less than 10.

The results of the linear regression model were significant [F(12,2806) = 281.99, p <.001, R^2^ = .55], indicating that approximately 55% of the variance in FCV-19S was explainable by gender, age, PHQ-9 and GAD-7. Specifically, the male category of gender significantly predicted the FCV-19S score [B = -0.76, t(2806) = -4.84, p <.001]. Based on this sample, this suggested that moving from the female to male gender category will decrease the mean value of FCV-19S score by 0.76 units on average. The 18-30 age category significantly predicted the FCV-19S score [B = -1.69, t(2806) = -9.13, p <.001]. Based on this sample, this suggested that moving from the 46–60 to the 18–30 age category will decrease the mean value of FCV-19S score by 1.69 units on average. The PHQ-9 score significantly predicted the FCV-19S score [B = -0.14, t(2806) = -6.24, p <.001]. This indicated that on average, a one-unit increase of PHQ-9 score will decrease the value of FCV-19S score by 0.14 units. Lastly, the GAD-7 score significantly predicted the FCV-19S score [B = 0.88, t(2806) = 39.72, p <.001]. This indicated that on average, a one-unit increase of GAD-7 score will increase the value of FCV-19S score by 0.88 units (**Table 10**).

**Table 10:** Linear regression with gender, age, education, PHQ-9, and GAD-7 predicting FCV-19S.

Variable	B	SE	CI	β	t	p
(Intercept)	8.16	0.30	[7.58, 8.75]	0.00	27.34	<.001
Gender = Male	-0.76	0.16	[-1.07, -0.45]	-0.06	-4.84	<.001
Age 31-45	-0.29	0.21	[-0.70, 0.11]	-0.02	-1.43	.153
Age 18-30	-1.69	0.19	[-2.05, -1.33]	-0.16	-9.13	<.001
Age 61-75	0.77	0.42	[-0.05, 1.59]	0.03	1.84	.065
Age > 75	2.18	1.24	[-0.26, 4.61]	0.02	1.76	.079
PHQ-9	-0.14	0.02	[-0.19, -0.10]	-0.12	-6.24	<.001
GAD-7	0.88	0.02	[0.84, 0.93]	0.79	39.72	<.001

CI is at the 95% confidence level; Results: F(12,2806) = 281.99, p <.001, R^2^ = .55; Unstandardized Regression Equation: FCV-19S = 8.16 - 0.76 * Gender = Male - 0.29 * Age 31–45 - 1.69 * Age 18–30 + 0.77 * Age 61–75 + 2.18 * Age > 75 -0.14 * PHQ-9 + 0.88 * GAD-7; FCV-19S, Fear of COVID-19 Scale; PHQ-9, Brief Patient Health Questionnaire Depression scale; GAD-7, Generalized Anxiety Disorder scale.

## Discussion

### Participants’ Sociodemographic Characteristics

The survey was distributed through the social media, including Facebook, by far the most popular social networking site in Greece (51, 52). Statistics on social media users in Greece indicated that the majority of users is between 25 and 44 years of age (e.g. Facebook users: 47.9% = 25–44 years old) (53) and lives in urban areas (54). The mean age of this sample was 35 years old, while roughly three out of four lived in an urban area.

Although the sex difference between male and female Greek users is not great (e.g. Facebook users: 52.7% = men; 47.3% = women) (53), the majority of this survey’s respondents were female. There is evidence of higher mental health literacy in women compared with men (55). Women are also more likely to participate in health-related online surveys, since they are more attracted to health topics and more active health-information seekers than men (56). Although there are no available Greek studies of mental health literacy and interest in health-related topics, evidence suggested that Greek women’s attitude towards mental health issues is altogether more positive than men’s (57). The survey’s headline, “Psychological burden related with the COVID-19 pandemic crisis”, may have attracted more female respondents due to its association with a health-related research purpose, a potential explanation for the abundancy of female responders.

Lastly, the majority of the respondents had a University Degree, something that may be related with the fact that Greece has the fourth highest tertiary enrolment rate among OECD countries, as well as high completion rates, that is, 81% for women and 74% for men (58).

### Potential Risk Factors for Increased Fear of COVID-19

Novel experiences tend to be more frightful (59). The SARS-CoV-2 is a novel virus with a rapid person-to-person transmission. The virus may also be transmitted from pre-symptomatic patients and potentially by asymptomatic carriers, a threat promoting fear (60). By April 30, the COVID-19 case fatality rate (CFR) was estimated at 7.25% worldwide and at 5.40% in Greece (61). The risk of severe COVID-19 in populations with defined risk factors was considered “very high”, while in the general population “moderate” (62 **).** Although research revealed risk factors for severe illness and death, including male gender, age over 65 years and underlying chronic medical conditions, the overall profile of high-risk patients has not been accurately defined yet (63). By April 30, 24.3% of COVID-19-related deaths in Greece involved patients aged between 40 and 64 years of age, while the NPHO representative highlighted that no one is immune to the disease (64). Taking COVID-19 lethality and unpredictability into account, contracting the virus, as well as worry about the health of relatives and friends may be risk factors for increased levels of COVID-19-related fear (32, 33). Therefore, this study explored two potential risk factors for increased fear of COVID-19, having contracted the virus, that is, personal experience with the disease, and having someone close with COVID-19, that is, worry about family members, friends, and significant others.

Nine respondents reported that they had contracted the virus, none belonged though to the high-risk group of people over 65 years of age. In addition, 68 participants had an affected member in their close environment. Interestingly, a significant proportion of participants, roughly one out of four, was not sure about whether they had been infected or not, whereas there were also respondents not knowing whether their relatives had been infected.

Since people with mental health disorders are likely to be more affected by COVID-19 due to higher vulnerability to stress compared with the general population (23, 34), this study considered a third potential risk factor for increased levels of fear, that is, receiving psychiatric medication during the past 6 months, indicative of the presence of a mental disease. Less than one out of 10 participants was on psychiatric medication. The majority was over the age of 46.

### Safety Behaviors

The fear of viral transmission during a pandemic is associated with precautionary measures, as well as avoidant behaviors to prevent infection. Preventive and mitigation measures include, among others, frequent hand hygiene, avoiding touching the face, wearing a medical mask in case of respiratory symptoms and keeping a minimum social distance of one meter. On March 19, WHO officially recommended a “rational use” of personal protective equipment to increase availability. For the healthy general population, emphasis was rather placed on hand hygiene and social distancing (65).

COVID-19 caused a “mask boom” worldwide. According to a Panhellenic survey conducted from March 17 to March 19 in 300 Greek pharmacies, products that had run out first were medical protective masks, antiseptics, disinfectant wipes, and alcohol solutions. In several areas, lack of masks and antiseptics had already been observed by the end of February (66). On April 9, a day before this study’s start date, the Greek Health Ministry representative drew attention to the fact that the use of masks and protective gloves may provide a false sense of security. As a result, the crucial protective measures, that is, social distance, and hand washing, may be overlooked. Therefore, the use of medical masks was not recommended for the healthy members of the Greek general population. Furthermore, there was a dispute over the use of plastic gloves due to lack of supportive evidence for their protective effects against the virus (67). Altogether, the benefit of mask usage for self-protection, as well as for preventing the spread of COVID-19 is an issue that remained controversial (68). Some experts in Greece continued to suggest precautionary use of masks in enclosed public places in case of even the slightest possibility that their use might prevent some of the virus’s transmission.

This study explored the employment of three safety behaviors, cleaning/disinfecting objects, taking care of personal hygiene and using personal protective equipment, such as face masks and disposable gloves. Almost half of the participants reported that they often clean/disinfect objects, while another 30% reported that they always do it. The majority of the respondents took care of personal hygiene according to the NPHO guidelines. Participants over the age of 46 tended to clean/disinfect objects and take care of personal hygiene more regularly compared with younger participants, while usage of personal protective equipment was more common at younger ages as well (over the age of 31).

### Checking Behaviors

A severe widespread disease such as COVID-19 may easily exacerbate health anxiety concerns. Fear for the body may cause hypervigilance, a state of alert with regard to physical changes suggesting COVID-19 illness (32). As a result, checking behaviors, such as body checking for symptoms and avoidance of potentially contaminated objects, may be employed (69). This study investigated three checking behaviors reflecting health anxiety, self-monitoring for COVID-19 symptoms, restricting physical contact with other people and communicating with family doctor due to fear of having been infected.

According to study findings, daily self-monitoring for COVID-19 symptoms was more frequent in participants aged between 61 and 75. On the contrary, the majority of participants over the age of 75 avoided monitoring themselves, possibly due to increased fear of COVID-19. In addition, the vast majority of participants over the age of 61, the high-risk group for severe illness, restricted physical contact with others.

Interestingly, around 90% of the participants across all ages had never contacted their family physician. At first glance, this finding may indicate that this checking behavior was limited. There may be though another explanation. Until now, less than one fifth of the Greek population has been registered with a family doctor, a form of primary health care that has been provided only recently in Greece. A shortage of such doctors may be related with confusion and is associated with increased emergency hospital visits in Greece. According to the recommendations of the NPHO, people with respiratory, COVID-19 resembling symptoms should avoid visiting a hospital, that is, people were advised against a common practice in case of health emergencies. Instead, they should contact their family doctor first, a less common emergency practice in Greece. Therefore, maybe less people contacted a family doctor, because access had not been easy. This is a hypothesis though that warrants further investigation (70, 71).

### Compliance With Guidelines and Social Responsibility

Compliance with guidelines by health authorities is a remedy for preventing the spread of the virus. Still, compliance, the “change in a person’s behavior in response to a direct request” may be more than just a behavioral response. Compliance may be influenced by personal beliefs, as well as personality characteristics. A study of a mixed sample from the United States, the United Kingdom, and Germany showed that maintenance of physical distance during the COVID-9 crisis, a compliant behavior, was promoted by empathy for high-risk members of the community (72). On the contrary, psychopathy was associated with non-compliance with health-related behaviors to restrict spread of COVID-19 (73).

The present study explored compliance by asking whether participants follow WHO guidelines and if they abide by the Greek government’s enacted measures. According to the results, half of the participants responded that they always comply with guidelines and over 40% that they often comply. Therefore, the proportion of the respondents who rarely or never comply was small (6.4%). Compliance with guidelines increased with age. In this sample, participants aged between 61 and 75 years displayed the most compliant behavior.

Social responsibility is an ethical concept. This study explored social responsibility motivation, that is, an individual’s motivation to act for the benefit of others. People with high social responsibility motivation do not only consider helping others a responsibility, they also want to help and enjoy supporting people and the community during crises. A study of adolescents in the US revealed that greater social responsibility was associated with less hoarding, a behavior leading to shortage of products, as well as regular use of antiseptics (74). In this sample, respondents within the age range 18–30 years showed the least social responsibility motivation. Roughly, half of the survey’s respondents belonged to this age group. Therefore, a potential explanation as to why some people overlook the health measures to restrict the spread of COVID-19 may be decreased social responsibility among younger members of the population, an observation that requires further investigation.

### The Psychological Impact of COVID-19

The first studies of the psychological impact of COVID-19 on the general population were conducted in China, where the virus first emerged. A cross-sectional study in a Chinese population 2 weeks after the outbreak reported a moderate to severe impact on more than half of study participants. Roughly one out of three participants displayed moderate to severe anxiety symptoms, while several others depressive symptoms and increased stress levels. The psychological impact was greater in women, students, people with physical symptoms resembling COVID-19 and people with poor self-reported health status. On the contrary, psychological symptoms were alleviated when precautionary health measures were taken, possible due to a better sense of control (75). Another cross-sectional study explored the psychological and behavioral responses in Chinese people from Wuhan and Shanghai again shortly after the outbreak. The study reported that the prevalence of moderate to severe anxiety increased by 4–5 times. Participants with confirmed or suspected COVID-19 cases around them displayed more severe anxiety symptoms than others. The majority of the respondents (over 90%) were compliant with guidelines and recommendations (76). Worry and concerns related with COVID-19 were reported in a Canadian (27), a US (28), and a German population (29). Altogether, the COVID-19 pandemic is expected to cause increased levels of anxiety, similarly to previous infectious disease outbreaks (77). Upcoming studies of the psychological impact of COVID-19 are awaited (78).

This study showed that 35.7% of the participants expressed high levels of COVID-19-related fear. Furthermore, 22.8% of the participants reported moderate to severe depressive symptoms, while a significant proportion, 77.4% of the respondents, reported moderate to severe anxiety symptoms. The rates of moderate to severe depressive and anxiety symptoms in this study were much higher than the rates reported in a Chinese population (16.5% and 28.8% respectively) (75). Apart from the different sociocultural background, perhaps previous experience with an epidemic may influence a general population’s reaction to the COVID-19 pandemic, explaining the different rates of severe depressive and anxiety symptoms between Greeks and Chinese. The last time Greece went through such a pandemic was a century ago, therefore the COVID-19 pandemic is a novel experience for the entire Greek population and novel experiences tend to be more frightful (59). On the contrary, China’s previous experience with the SARS outbreak was more recent. Notably, in the beginning of February, WHO announced the name “COVID-19” for the SARS-CoV-2 virus disease. This name predominated in public communication worldwide to protect the population, especially Asians, from collective memories of the SARS outbreak in 2003 (79). Whether previous experience with other pandemics may ameliorate or increase the psychological burden of the current experience with COVID-19 remains to be evaluated.

Women showed significantly higher levels of COVID-19-related fear, as well as more severe anxiety symptoms, compared with men. This finding is in accordance with evidence suggesting that women report more fear and anxiety than men (80). In addition, women showed significantly more severe depressive symptoms than men. Based on the fact that depression is more common in Greek women, that is, seven out of ten depressive patients are female (81), it was expected that females would demonstrate more severe depressive symptoms than men in this sample. Altogether, this study observed a greater psychological burden in female compared with male responders. This observation is in accordance with findings of other studies, for instance a study of a German population that reported greater worry about COVID-19 in women compared with men (29), as well as a study of a Chinese population that revealed a greater psychological impact of COVID-19 on women, that is, higher levels of stress, anxiety, and depression, compared with men (75).

This study confirmed the significant positive correlations between fear of COVID-19, depressive, and anxiety symptoms reported previously (31). With regard to age, younger participants, under the age of 30, displayed less fear of COVID-19 and reported less severe depressive symptoms compared with older participants. Contrary to fear and depressive symptoms, anxiety symptoms’ severity in younger participants did not differ significantly compared with older ones.

### Fear of COVID-19

Infectious disease outbreaks evoke automatically a fear response, an alert state, possibly related with the collective memory of past deadly pandemics (24). Similarly, a significant psychological effect of the COVID-19 pandemic crisis is fear (82). People with mental diseases are more vulnerable to stress compared with the general population and are therefore more likely to be affected by COVID-19. As expected, this study revealed that being on psychiatric medication was associated with higher levels of COVID-19-related fear (23, 34).

Fear is considered a biologically “basic” emotion (83), an automatic “reflex” response to a specific external danger. There is a great overlap between fear and anxiety, the latter being more related with an unknown, vague or upcoming threat. From an evolutionary perspective, fear is associated with risk-avoiding behaviors, while anxiety with preparedness. Both emotional states promote adaptation and self-perseverance (35). Since fear is associated with self-perseverance, it may elicit checking behaviors (32) and promote self-protective responses (84). A recent study assessed fear of COVID-19 by the FCV-19S scale in 324 participants from the United Kingdom. According to the results, participants showing higher levels of fear employed more public health behaviors. Therefore, the authors concluded that this “functional” form of fear could be carefully used by health authorities to nurture safety behaviors (30). Still, it should be noted that fear does not only promote self-protective behaviors, but also defensive responses to control fear, such as denial (e.g., “I am not at risk of becoming infected”). These two competing responses are inversely correlated, that is, the more one remains in denial or defensive avoidance rejecting fear, the less one follows recommendations to escape the actual danger. Moreover, amplifying fear may promote defensive responses, rather than self-protective actions, particularly in individuals with weaker self-efficacy perceptions, that is, lower perceived ability to perform a self-protective behavior (84). Therefore, although fear has been used to address public health issues and to promote health education, definite beneficial effects of such an approach have not always been demonstrated (85, 86). Fear is effective in producing behavioral changes only when it is accompanied by high-efficacy messages, e.g. concrete references to the severity of a disease and/or the susceptibility to it (84).

Vice versa, although individuals employ safety behaviors to shield themselves from danger and harm, safety behaviors may intensify fear and anxiety. A study of undergraduate participants with different levels of contamination fear revealed that enforcement of daily safety behaviors raised awareness of contamination, increasing contamination-related fear. Worry of contamination increased in all participants, regardless of the initial levels of contamination fear (87). Except for contamination fear, safety behaviors were also associated with higher levels of health anxiety (88). Moreover, it is well known that safety and avoidant behaviors related with anxiety disorders, as well as control behaviors related with obsessive compulsive disorder, amplify fear and anxiety respectively (89). In accordance, this study showed that employment of safety behaviors, as well as checking behaviors, that is, self-monitoring for COVID-19 symptoms and communication with the family doctor, were associated with higher levels of fear. On the contrary, restricting physical contact with others was not related with fear. This finding may be explained by the fact that although personal experience with the virus was not associated with fear, a close person’s COVID-19 illness was related with higher levels of fear. Taken together, it could be hypothesized that restricting physical contact was not associated with fear because this checking behavior protects family members and friends by securing social distancing. In addition, greater compliance with WHO guidelines and the measures that the government has enacted was also associated with increased levels of fear. Personal safety measures and self-monitoring behaviors are incorporated in WHO guidelines, in such, increased compliance with WHO may amplify fear. The regular controls by the Hellenic Police, the Municipal police and the Hellenic Coast Guard, deployed by the Greek government to ensure abidance to the restriction measures, potentially reinforced fear as well. Altogether, this study provided evidence that excessive behavioral responses to the pandemic amplify fear of COVID-19. Therefore, a vicious circle may be created between excessive behavioral responses and increased fear of COVID-19.

Based on previous experience with infectious disease outbreaks, fear may be related with various psychological factors. A recent study reported health anxiety and intolerance of uncertainty to be predictors of fear of coronavirus (33). This study showed that gender, age, depressive and anxiety symptoms modified levels of fear of COVID-19. Specifically, female gender, older age, as well as more severe anxiety symptoms indicated higher levels of COVID-19-related fear. These findings are interpretable, taking into account the fact that women report more fear than men (80), greater age is associated with a higher prevalence of underlying risk conditions for severe COVID-19 disease and mortality (90) and anxiety overlaps with fear (35). On the contrary, more severe depressive symptoms indicated lower levels of COVID-19-related fear. This finding is interpretable, taking into account the fact that depressive patients display reduced emotional responsiveness and aloof responses (91). Therefore, individuals with more severe depressive symptoms may remain indifferent towards the threat of COVID-19, experiencing less fear.

### Study’s Usefulness and Limitations

A pandemic is a public health emergency situation. Pandemics are not only life-threatening conditions; they have an impact on mental health. The magnitude of the psychosocial impact during the COVID-19 pandemic, as well as the long-term consequences, may be affected by personal, socio-cultural and other situational factors, as well as by a country’s economy. Due to the globalization, studies of a variety of populations across different countries are necessary to gain an overall understanding of the impact of COVID-19 to implement management strategies. Up to date, there are no available studies of the psychosocial impact of previous epidemics on the Greek population. Moreover, to the best of our knowledge, this was the first study exploring fear of COVID-19 in a Greek population. The study was conducted as soon as 3 weeks after a national lockdown had been imposed to explore fear during the initial phase of this novel experience. Studies of fear during a pandemic, describing the sociodemographic factors associated with fear together with other modulators, enable the creation of both supportive and preventive interventions, targeting risk groups.

Still, the present study had some limitations: i) the study’s cross-sectional design did not permit the elucidation of causal relationships (92); ii) the results were obtained based on self-report information and self-administered tools, and may therefore suffer from bias (93); iii) the respondents’ characteristics with regard to occupational status were not described in detail (lack of data on number of students and retirees). In addition, the questions exploring safety behaviors did not consider potential lack of information about NPHO health guidelines; iv) due to the strict restriction measures, participants completed online survey questionnaires. Although there is evidence supporting the view that online data collection is equivalent to paper-and-pencil data collection (94, 95), online surveys remain subjected to criticism with regard to data quality; v) although an online survey may provide a large amount of data within a short period, the internet community may not be representative of the general population, as older, less educated and socially disadvantaged groups may not be adequately represented. In addition, online surveys suffer from the so-called “volunteer-effect”, associated with the fact that people respond to surveys when they are interested in the topic or when they identify themselves with the survey’s scope. Therefore, bias associated with self-selection cannot be excluded, since responders’ characteristics may differ substantially from non-responders, limiting results’ generalizability (56).

## Conclusions

A pandemic is a dread risk, a relatively rare event causing death to many people within a short period of time. People respond with more fear and anxiety to dread risks compared with continuous risks, affecting people over a longer period (96). This study investigated the psychological impact of COVID-19 in Greece during the initial phase of the pandemic, as well as the complex associations between fear of COVID-19, depressive symptoms, anxiety symptoms, potential risk factors for increased COVID-19-related fear, safety behaviors, checking behaviors, compliance with guidelines and social responsibility. Based on this study’s results, excessive safety, and checking behaviors, as well as greater compliance with guidelines may amplify fear, potentially due to increased contamination awareness. Therefore, this study underscores the danger of a vicious circle’s creation between fear of COVID-19 and behavioral responses to the pandemic. Furthermore, female gender, older age, as well as more severe depressive and anxiety symptoms were shown to modulate levels of COVID-19-related fear.

Meanwhile, a brief mental health screening questionnaire for COVID-19-related anxiety was developed, the Coronavirus Anxiety Scale (CAS), not available when this study was conducted (97). Specific psychometric tools, such as the FCV-19S and CAS scales, may support future studies. Additional studies are required to describe psychologically vulnerable groups with regard to religiosity, personality traits and other factors (82). Future studies, focusing on groups that are more vulnerable to the pandemic-related stress, such as the COVID-19 patients, victims’ relatives, people with medical conditions, psychiatric patients and health-care professionals, are also of great importance (23).

Conclusively, since previous experience with the SARS epidemic in 2003 yielded evidence that outbreaks of infectious diseases may be associated with long-lasting consequences on mental health (98), the long-term impact of COVID-19 remains to be evaluated.

## Data Availability Statement

The raw data supporting the conclusions of this article will be made available by the authors, without undue reservation.

## Ethics Statement

This study involving human participants was reviewed by the Scientific Committee of the General Hospital “Papageorgiou” Review Board, Thessaloniki, Greece. Ethical approval was received prior to data collection. The study was anonymous. Before entering the online-survey, respondents were requested to indicate their consent. To secure anonymity, the selection “anonymize responses” chosen on the online platform prevented recording of any personal information and assured removal of all contact associations.

## Author Contributions

EP and V contributed equally to this study. EP contributed to intellectual input and wrote the first draft of the manuscript. VH contributed to study’s conception and design, as well as to data management interpretation. PV, AB, IG, AG, and GP contributed to literature search and paper editing. KaP, AD, AC, VB, SP, MS, KP, and CK contributed to study’s design, creation, and distribution of the online survey and data management. ID supervised the study and contributed to the final revision of the manuscript. All authors contributed to the article and approved the submitted version.

## Conflict of Interest

The authors declare that the research was conducted in the absence of any commercial or financial relationships that could be construed as a potential conflict of interest.

## References

[1] World Health Organization (WHO) Novel coronavirus-China. Jan 12, 2020. World Health Organization (2020). Retrieved from http://www.who.int/csr/don/12-january-2020-novel-coronavirus-china/en/.

[2] World Health Organization (WHO) Novel coronavirus-Thailand (ex-China). Jan 14, 2020. World Health Organization (2020). Retrieved from http://www.who.int/csr/don/14-january-2020-novel-coronavirusthailand/en/.

[3] Holshue M DeBolt C Lindquist S Lofy KH Wiesman J Bruce H First case of 2019 novel coronavirus in the United States. N Engl J Med (2020) 382:929–36. 10.1056/NEJMoa2001191 PMC7092802 32004427

[4] World Health Organization (WHO) 2019-nCoV outbreak is an emergency of international concern. Jan 31, 2020. World Health Organization (2020). Retrieved from http://www.euro.who.int/en/health-topics/health-emergencies/coronavirus-covid-19/news/news/2020/01/2019-ncov-outbreak-is-an-emergency-of-international-concern.

[5] World Health Organization (WHO) WHO Coronavirus Disease (COVID-19) Dashboard. World Health Organization (2020). Retrieved from https://covid19.who.int/.

[6] World Health Organization (WHO) WHO announces COVID-19 disease outbreak a pandemic. Mar 12, 2020. World Health Organization (2020), Retrieved from http://www.euro.who.int/en/health-topics/health-emergencies/coronavirus-covid-19/news/news/2020/3/who-announces-covid-19-outbreak-a-pandemic.

[7] National Public Health Organization (NPHO) Current state of Covid-19 outbreak in Greece and timeline of key containment events, March 4, 2020. National Public Health Organization (2020), Retrieved from https://eody.gov.gr/en/current-state-of-covid-19-outbreak-in-greece-and-timeline-of-key-containment-events/.

[8] Naftemporiki Covid-19 crisis: Greek government details measures, exclusions for unprecedented ban on public movement. Naftemporiki [Internet] (2020), https://www.naftemporiki.gr/story/1578668/covid-19-crisis-greek-govt-details-measures-exclusions-for-unprecendeted-ban-on-public-movement.

[9] National Public Health Organization (NPHO) Novel coronavirus COVID-19-Guidelines. National Public Health Organization (2020). Retrieved from https://eody.gov.gr/neos-koronaios-covid-19/.

[10] National Public Health Organization (NPHO) Guidance on self-isolation at home, March 13, 2020. National Public Health Organization (2020). Retrieved from https://eody.gov.gr/en/guidance-on-self-isolation-at-home/.

[11] Naftemporiki Greek government extends temporary coronavirus restrictions to May 4. Naftemporiki [Internet] (2020). Retrieved from https://www.naftemporiki.gr/story/1592379/greek-govt-extends-temporary-coronavirus-restrictions-to-may-4.

[12] Bonovas S Nikolopoulos G High-burden epidemics in Greece in the era of economic crisis. Early signs of a public health tragedy. J Prev Med Hyg (2012) 53(3):169–71. 10.15167/2421-4248/jpmh2012.53.3.340 23362624

[13] World Health Organization (WHO) Summary of probable SARS cases with onset of illness from 1 November 2002 to 31 July 2003. World Health Organization (2003). Retrieved from https://www.who.int/csr/sars/country/table2004_04_21/en/.

[14] Athanasiou M Lytras T Spala G Triantafyllou E Gkolfinopoulou K Theocharopoulos G Fatal cases associated with pandemic influenza A (H1N1) reported in Greece. PloS Curr (2010) 2:RRN1194. 10.1371/currents.RRN1194 21085493 PMC2976846

[15] Centers for Disease Control and Prevention (CDC) Deaths and Hospitalizations Related to 2009 Pandemic Influenza A (H1N1)-Greece, May 2009-February 2010. June 11, 2010. Centers for Disease Control and Prevention (2010) 59(22):682–6, Retrieved from https://www.cdc.gov/mmwr/preview/mmwrhtml/mm5922a2.htm. 20535092

[16] National Public Health Organization (NPHO) Epidemiological Report (COVID-19), April 27, 2020. National Public Health Organization (2020). Retrieved from https://eody.gov.gr/wp-content/uploads/2020/04/covid-gr-daily-report-20200427.pdf.

[17] Goulia P Mantas C Dimitroula D Mantis D Hyphantis T General hospital staff worries, perceived sufficiency of information and associated psychological distress during the A/H1N1 influenza pandemic. BMC Infect Dis (2010) 10:322. 10.1186/1471-2334-10-322 21062471 PMC2990753

[18] Tsoucalas G Sgantzos M The 2009 influenza A virus-subtype H1N1 pandemic, a glance from Greece. Le Infezioni Med (2016) 4:259–64, Available from www.jstor.org/stable/44447656 28011959

[19] Patterson KD Pyle GF The geography and mortality of the 1918 influenza pandemic. Bull History Med (1991) 65(1):4–21. 2021692

[20] Mammas IN Theodoridou M Spandidos DA The 1918 Spanish flu outbreak that devastated a Greek island underlines past lessons that must never be forgotten. Acta Paediatr (2018) 107(11):2034. 10.1111/apa.14351 29604107

[21] Tsoucalas G Karachaliou F Kalogirou V Gatos G Mavrogiannaki E Antonios Antoniou A The first announcement about the 1918 “Spanish Flu” pandemic in Greece through the writings of the pioneer newspaper “Thessalia” almost a century ago. Infez Med (2015) 23(1):79–82. 25819057

[22] Pappas S How will people react to the new financial crisis? American Psychological Association (2020), Retrieved from https://www.apa.org/news/apa/2020/04/financial-crisis-covid-19.

[23] Pan American Health Organization (PAHO/WHO) Protecting mental health during epidemics. American Health Organization (2009). Retrieved from https://www.paho.org/en/documents/protecting-mental-health-during-epidemics.

[24] Van Damme W Van Lerberghe W Epidemics and fear. Trop Med Int Health (2000) 5(8):511–4. 10.1046/j.1365-3156.2000.00599.x 10995091

[25] Pappas G Seitaridis S Akritidis N Tsianos E Infectious diseases in cinema: virus hunters and killer microbes. Clin Infect Dis (2003) 37:939–42. 10.1086/377740 13130406

[26] Tomes N The making of a germ panic, then and now. Am J Public Health (2000) 90(2):191–8. 10.2105/ajph.90.2.191 PMC1446148 10667179

[27] Asmundson GJG Taylor S Coronaphobia: Fear and the 2019-nCoV outbreak. J Anxiety Disord (2020) 70:102196. 10.1016/j.janxdis.2020.102196 32078967 PMC7134790

[28] McCarthy J U.S. coronavirus concerns surge, Government trust slides. Gallup (2020). Retrieved from https://news.gallup.com/poll/295505/coronavirus-worries-surge.aspx.

[29] Gerhold L COVID-19: Risk perception and coping strategies. Results from a survey in Germany. Manuscript submitted for publication. PsyArXiv Preprints. (2020) [preprint]. 10.31234/osf.io/xmpk4

[30] Harper CA Satchell LP Fido D Latzman RD Functional fear predicts public health compliance in the COVID-19 pandemic. PsyArXiv Preprints. (2020) 1–14. 10.1007/s11469-020-00281-5 PMC7185265 32346359

[31] Ahorsu DK Lin CY Imani V Saffari M Griffiths MD Pakpour AH The Fear of COVID-19 Scale: Development and Initial Validation. Int J Ment Health Addict (2020), 1–9. 10.1007/s11469-020-00270-8 PMC7100496 32226353

[32] Schimmenti A Billieux J Starcevic V The four horsemen of fear: An integrated model of understanding fear experiences during the COVID-19 pandemic. Clin Neuropsychiat (2020) 17(2):41–5. 10.36131/CN20200202 PMC8629088 34908966

[33] Mertens G Gerritsen L Duijndam S Salemink E Engelhard IM Fear of the coronavirus (COVID-19): Predictors in an online study conducted in March 2020. J Anxiety Disord (2020) 74:102258 10.1016/j.janxdis.2020.102258 32569905 PMC7286280

[34] Yao H Chen J-H Xu Y-F Patients with mental health disorders in the COVID-19 epidemic. Lancet Psychiatry (2020) 7(4):e21. 10.1016/S2215-0366(20)30090-0 32199510 PMC7269717

[35] Steimer T The biology of fear- and anxiety-related behaviors. Dialogues Clin Neurosci (2002) 4(3):231–49. 10.31887/DCNS.2002.4.3/tsteimer PMC3181681 22033741

[36] Cheng C Tang CS The psychology behind the masks: psychological responses to the severe acute respiratory syndrome outbreak in different regions. Asian J Soc Psychol (2004) 7:3–7. 10.1111/j.1467-839X.2004.00130.x 32313436 PMC7159619

[37] Vartti AM Oenema A Schreck M Uutela A de Zwart O Brug J SARS knowledge, perceptions, and behaviors: a comparison between Finns and the Dutch during the SARS outbreak in 2003. Int J Behav Med (2009) 16:41–8. 10.1007/s12529-008-9004-6 PMC7091200 19184625

[38] QualtricsXM Support. (2020). Available at: https://www.qualtrics.com/support/survey-platform/getting-started/survey-platform-overview/ (Accessed April 2, 2020).

[39] Bradburn NM Seymour S Brian W Asking Questions: The Definitive Guide to Questionnaire Design-For Market Research, Political Polls, and Social and Health Questionnaires, Revised Edition. Jossey-Bass: San Francisco (2004).

[40] Qualtrics Experience Management Software. (2020). Available at https://www.qualtrics.com/.

[41] Tsipropoulou V Nikopoulou VA Holeva V Nasika Z Diakogiannis I Sakka S Psychometric properties of the Greek version of FCV19-S. Int J Ment Health Addict (2020) 1–10. 10.1007/s11469-020-00319-8 PMC7250285 32837420

[42] Nikopoulou VA Holeva V Parlapani E Karamouzi P Voitsidis P Porfyri GN Mental health screening for COVID-19: A proposed cut-off score for the Fear of COVID-19 Scale (FCV-19S). Int J Ment Health Addict (2020). in press. 10.1007/s11469-020-00414-w PMC7654349 33199975

[43] Kroenke K Spitzer RL Williams JBW The PHQ-9 Validity of a Brief Depression Severity Measure. J Gen Intern Med (2001) 16(9):606–13. 10.1046/j.1525-1497.2001.016009606.x PMC1495268 11556941

[44] Martin A Rief W Klaiberg A Braehler E Validity of the Brief Patient Health Questionnaire Mood Scale (PHQ-9) in the General Population. Gen Hosp Psychiatry (2006) 28(1):71–7. 10.1016/j.genhosppsych.2005.07.003 16377369

[45] The Patient Health Questionnaire (PHQ) Screeners Instruction manual. (2020). Retrieved from https://www.phqscreeners.com/images/sites/g/files/g10016261/f/201412/instructions.pdf (Accessed March 29, 2020).

[46] The Patient Health Questionnaire (PHQ) Screeners Greek version of PHQ-9. (2020). Retrieved from https://www.phqscreeners.com/images/sites/g/files/g10060481/f/201412/PHQ9_Greek%20for%20Greece.pdf (Accessed March 29, 2020).

[47] Spitzer RL Kroenke K Williams JBW Löwe B A brief measure for assessing generalized anxiety disorder: The GAD-7. Arch Intern Med (2006) 166(10):1092–7. 10.1001/archinte.166.10.1092 16717171

[48] The Patient Health Questionnaire (PHQ) Screeners Greek version of GAD-7. (2020). Retrieved from https://www.phqscreeners.com/images/sites/g/files/g10060481/f/201412/GAD7_Greek%20for%20Greece.pdf (Accessed March 29, 2020).

[49] Steele W Schreiber G Guiltinan A Nass C Glynn S Wright D The role of altruistic behavior, empathetic concern, and social responsibility motivation in blood donation behavior. Transfusion (2008) 48(1):43–54. 10.1111/j.1537-2995.2007.01481.x 17894795

[50] IBM Corp Released 2017. IBM SPSS Statistics for Windows, Version 26.0. IBM Corp: Armonk, NY (2017).

[51] Statcounter Social Media Stats Greece, April 2019-April 2020. StatCounter GlobalStats (2020). Retrieved from https://gs.statcounter.com/social-media-stats/all/greece.

[52] Drosos D Tsotsolas N Chalikias M Skordoulis M Koniordos M A survey on the use of social networking sites in Greece. In: Kravets A Shcherbakov M Kultsova M Shabalina O, editors. Creativity in Intelligent Technologies and Data Science. Communications in Computer and Information Science, vol. 535 Cham: Springer (2015). p. 556–70.

[53] NapoleonCat Stats Social media users in Greece, March 2020. NapoleonCat Stats (2020). Retrieved from https://napoleoncat.com/stats/social-media-users-in-greece/2020/03.

[54] Papadakis M Greece: Profiling of the users of social media by FOCUS BARI Research. Mediarisk (2015). Retrieved from http://www.mediarisk.gr/greece-profiling-of-the-users-of-social-media-by-focus-bari-research/.

[55] Furnham A Swami V Mental health literacy: A review of what it is and why it matters. Int Perspect Psychol: Res Practice Consult (2018) 7(4):240–57. 10.1037/ipp0000094

[56] Eysenbach G Wyatt J Using the internet for surveys and health research. J Med Internet Res (2002) 4(2):E13. 10.2196/jmir.4.2.e13 12554560 PMC1761932

[57] Efstathiou G Kouvaraki E Ploubidis G Kalantzi-Azizi A Self-stigma, public-stigma and attitudes towards professional psychological help: Psychometric properties of the Greek version of three relevant questionnaires. Int J Adv Counsel (2019) 41(2):175–86. 10.1007/s10447-018-9364-9

[58] OECD/UIS/Eurostat Education at a glance 2019, Greece. Organization for Economic Co-operation and Development (2019). Retrieved from https://gpseducation.oecd.org/Content/EAGCountryNotes/GRC.pdf.

[59] Ropeik D The consequences of fear. EMBO Rep (2004) 5(Suppl 1):S56–60. 10.1038/sj.embor.7400228 PMC1299209 15459737

[60] World Health Organization (WHO) Coronavirus disease 2019 (COVID-19) Situation Report-73. Apr 2, 2020. World Health Organization (2020). Retrieved from https://www.who.int/docs/default-source/coronaviruse/situation-reports/20200402-sitrep-73-covid-19.pdf?sfvrsn=5ae25bc7_2.

[61] Our World in Data Case fatality rate of the ongoing COVID-19 pandemic. Source: European CDC-Situation Update Worldwide; published online at OurWorldInData.org (2020). Retrieved from https://ourworldindata.org/grapher/coronavirus-cfr?country=OWID_WRL+GRC.

[62] European Centre for Disease Prevention and Control (ECDC) Risk assessment on COVID-19. Apr 8, 2020. European Centre for Disease Prevention and Control (2020). Retrieved from https://www.ecdc.europa.eu/en/current-risk-assessment-novel-coronavirus-situation. 10.2807/1560-7917.ES.2020.25.8.2002271 PMC7055041 32127126

[63] Zheng Z Peng F Xu B Zhao J Liu H Peng J Risk factors of critical & mortal COVID-19 cases: A systematic literature review and meta-analysis. J Infect (2020). 81(2):e16–e25. 10.1016/j.jinf.2020.04.021 PMC7177098 32335169

[64] National Public Health Organization (NPHO) Epidemiological Report (COVID-19), April 30, 2020. National Public Health Organization (2020). Retrieved from https://eody.gov.gr/wp-content/uploads/2020/04/covid-gr-daily-report-20200430.pdf.

[65] World Health Organization (WHO) Rational use of personal protective equipment (PPE) for coronavirus disease (COVID-19). Interim guidance, March 19, 2020. World Health Organization (2020). Retrieved from https://apps.who.int/iris/bitstream/handle/10665/331498/WHO-2019-nCoV-IPCPPE_use-2020.2-eng.pdf.

[66] The News Coronavirus: Pharmacy shortage of products. March 24, 2020. Ta Nea Online [Internet] (2020). Retrieved from https://www.tanea.gr/2020/03/24/greece/koronaios-deite-poia-proionta-parousiazoun-elleipsi-sta-farmakeia/.

[67] National Public Health Organization (NPHO) COVID-19 Announcement, April 9, 2020. National Public Health Organization (2020). Retrieved from https://eody.gov.gr/0409_briefing_covid19/.

[68] Greenhalgh T Schmid MB Czypionka T Bassler D Gruer L Face masks for the public during the covid-19 crisis. BMJ (2020) 369:m1435. 10.1136/bmj.m1435 32273267

[69] Smith A Hypochondria and COVID-19: Health anxiety and coronavirus. Medical News Today (2020). Retrieved from https://www.medicalnewstoday.com/articles/hypochondria-and-covid-19.

[70] Adamakidou T Kalokerinou A New health policies on Primary Health Care in Greece. Health Sci J (2010) 4(1):15–23.

[71] Mpouloutza P The government’s breakthrough in Health care is a “great patient”. April 23, 2020. Kathimerini [Internet] (2020). Retrieved from https://www.kathimerini.gr/1030339/article/epikairothta/ellada/megalos-as8enhs-h-kyvernhtikh-tomh-sthn-ygeia.

[72] Pfattheicher S Nockur L Böhm R Sassenrath C Petersen MB The emotional path to action: Empathy promotes physical distancing during the COVID-19 pandemic. PsyArXiv Preprints (2020). 10.31234/osf.io/y2cg5 32993455

[73] Blagov PS Adaptive and dark personality traits in the COVID-19 pandemic: Predicting health-behavior endorsement and the appeal of public health messages. Soc Psychol Personal Sci (2020) 1–11. 10.1177/1948550620936439 PMC7342937 38602980

[74] Oosterhoff B Palmer C Psychological correlates of news monitoring, social distancing, disinfecting, and hoarding behaviors among US adolescents during the COVID-19 pandemic. PsyArXiv Preprints. (2020). 10.31234/osf.io/rpcy4 PMC7325067 32597925

[75] Wang C Pan R Wan X Tan T Xu L Ho CS Immediate psychological responses and associated factors during the initial stage of the 2019 Coronavirus Disease (COVID-19) Epidemic Among the General Population in China. Int J Environ Res Public Health (2020) 17(5):1729. 10.3390/ijerph17051729 PMC7084952 32155789

[76] Qian M Wu Q Wu P Hou Z Liang Y Cowling BJ Psychological responses, behavioral changes and public perceptions during the early phase of the COVID-19 outbreak in China: a population based cross-sectional survey. medRxiv Preprints. (2020). 10.1101/2020.02.18.20024448 PMC7545627 33033099

[77] Fardin MA COVID-19 and Anxiety: A review of psychological impacts of infectious disease outbreaks. Arch Clin Infect Dis (2020) e102779. 10.5812/archcid.102779 in press.

[78] Rajkumar RP COVID-19 and mental health: A review of the existing literature. Asian J Psychiatr (2020) 52:102066. 10.1016/j.ajp.2020.102066 32302935 PMC7151415

[79] World Health Organization (WHO) Naming the coronavirus disease (COVID-19) and the virus that causes it. World Health Organization (2020). Retrieved from https://www.who.int/emergencies/diseases/novel-coronavirus-2019/technical-guidance/naming-the-coronavirus-disease-(covid-2019)-and-the-virus-that-causes-it.

[80] McLean CP Anderson ER Brave men and timid women? A review of the gender differences in fear and anxiety. Clin Psychol Rev (2009) 29(6):496–505. 10.1016/j.cpr.2009.05.003 19541399

[81] Hellenic Statistical Authority Health Interview Survey 2014. Announcement: Piraeus, June 10, 2016. Hellenic Statistical Authority (2016).

[82] Pakpour AH Griffiths MD The fear of COVID-19 and its role in preventive behaviors. J Concurr Disord (2020). Retrieved from https://concurrentdisorders.ca/2020/04/03/the-fear-of-covid-19-and-its-role-in-preventive-behaviors/.

[83] Adolphs R The Biology of Fear. Curr Biol (2013) 23(2):R79–93. 10.1016/j.cub.2012.11.055 PMC3595162 23347946

[84] Witte K Allen M A meta-analysis of fear appeals: Implications for effective public health campaigns. Health Educ Behav (2000) 27(5):591–615. 10.1177/109019810002700506 11009129

[85] Pappas G Kiriaze IJ Giannakis P Falagas ME Psychosocial consequences of infectious diseases. Clin Microbiol Infect (2009) 15(8):743–7. 10.1111/j.1469-0691.2009.02947.x PMC7129378 19754730

[86] Nabi RL Myrick JG Uplifting fear appeals: Considering the role of hope in fear-based persuasive messages. Health Commun (2019) 34(4):463–74. 10.1080/10410236.2017.1422847 29313717

[87] Deacon B Maack DJ The effects of safety behaviors on the fear of contamination: An experimental investigation. Behav Res Ther (2008) 46(4):537–47. 10.1016/j.brat.2008.01.010 18313031

[88] Olatunji BO Etzel EN Tomarken AJ Ciesielski BG Deacon B The effects of safety behaviors on health anxiety: An experimental investigation. Behav Res Ther (2011) 49(11):719–28. 10.1016/j.brat.2011.07.008 21839987

[89] Helbig-Lang S Petermann F Tolerate or Eliminate? A systematic review on the effects of safety behavior across anxiety disorders. Clin Psychol Sci Pract (2010) 17(3):218–33. 10.1111/j.1468-2850.2010.01213.x

[90] Clark A Jit M Warren-Gash C Guthrie B Wang HHX Mercer SW How many are at increased risk of severe COVID-19 disease? Rapid global, regional and national estimates for 2020. medRxiv Preprints. (2020). 10.1101/2020.04.18.20064774

[91] Kreuch G Stephan and Slaby’s complementary work. In: Kreuch G, editor. Self-Feeling: Can self-consciousness be understood as a feeling? Switzerland: Springer Nature (2019). p. 102–4.

[92] Mann CJ Observational research methods. Research design II: cohort, cross sectional, and case-control studies. Emerg Med J (2003) 20:54–60. 10.1136/emj.20.1.54 12533370 PMC1726024

[93] Podsakoff PM MacKenzie SB Lee JY Podsakoff NP Common method biases in behavioral research: a critical review of the literature and recommended remedies. J Appl Psychol (2003) 88:879–903. 10.1037/0021-9010.88.5.879 14516251

[94] Davidov E Depner F Testing for measurement equivalence of human values across online and paper-and-pencil surveys. Qual Quant (2011) 45:375–90. 10.1007/s11135-009-9297-9

[95] Weigold A Weigold IK Russell EJ Examination of the equivalence of self-report survey-based paper-and-pencil and internet data collection methods. Psychol Methods (2013) 18:53–70. 10.1037/a0031607 23477606

[96] Bodemer N Ruggeri A Galesic M When dread risks are more dreadful than continuous risks: Comparing cumulative population losses over time. PloS One (2013) 8(6):e66544. 10.1371/journal.pone.0066544 23840503 PMC3694073

[97] Lee SA Coronavirus Anxiety Scale: A brief mental health screener for COVID-19 related anxiety. Death Stud (2020) 16:1–9. 10.1080/07481187.2020.1748481 32299304

[98] Liua X Kakadeb M Fullerb CJ Fanb B Fangc Y Kongc J Depression after exposure to stressful events: Lessons learned from the SARS epidemic. Compr Psychiatry (2012) 53(1):15–23. 10.1016/j.comppsych.2011.02.003 21489421 PMC3176950

